# Increased plasma disequilibrium between pro- and anti-oxidants during the early phase resuscitation after cardiac arrest is associated with increased levels of oxidative stress end-products

**DOI:** 10.1186/s10020-021-00397-x

**Published:** 2021-10-24

**Authors:** Muhammad Shoaib, Nancy Kim, Rishabh C. Choudhary, Tai Yin, Koichiro Shinozaki, Lance B. Becker, Junhwan Kim

**Affiliations:** 1 Donald and Barbara Zucker School of Medicine at Hofstra/Northwell, Hempstead, NY USA; 2 Laboratory for Critical Care Physiology, The Feinstein Institutes for Medical Research, 350 Community Dr., Manhasset, NY 11030 USA; 3 Department of Emergency Medicine, Northwell Health, NY USA

**Keywords:** Cardiac arrest, Oxidative stress, Reactive oxygen species, Prooxidants, Antioxidant disequilibrium

## Abstract

**Background:**

Cardiac arrest (CA) results in loss of blood circulation to all tissues leading to oxygen and metabolite dysfunction. Return of blood flow and oxygen during resuscitative efforts is the beginning of reperfusion injury and is marked by the generation of reactive oxygen species (ROS) that can directly damage tissues. The plasma serves as a reservoir and transportation medium for oxygen and metabolites critical for survival as well as ROS that are generated. However, the complicated interplay among various ROS species and antioxidant counterparts, particularly after CA, in the plasma have not been evaluated. In this study, we assessed the equilibrium between pro- and anti-oxidants within the plasma to assess the oxidative status of plasma post-CA.

**Methods:**

In male Sprague–Dawley rats, 10 min asphyxial-CA was induced followed by cardiopulmonary resuscitation (CPR). Plasma was drawn immediately after achieving return of spontaneous circulation (ROSC) and after 2 h post-ROSC. Plasma was isolated and analyzed for prooxidant capacity (Amplex Red and dihydroethidium oxidation, total nitrate and nitrite concentration, xanthine oxidase activity, and iron concentration) and antioxidant capacity (catalase and superoxide dismutase activities, Total Antioxidant Capacity, and Iron Reducing Antioxidant Power Assay). The consequent oxidative products, such as 4-Hydroxyl-2-noneal, malondialdehyde, protein carbonyl, and nitrotyrosine were evaluated to determine the degree of oxidative damage.

**Results:**

After CA and resuscitation, two trends were observed: (1) plasma prooxidant capacity was lower during ischemia, but rapidly increased post-ROSC as compared to control, and (2) plasma antioxidant capacity was increased during ischemia, but either decreased or did not increase substantially post-ROSC as compared to control. Consequently, oxidation products were increased post-ROSC.

**Conclusion:**

Our study evaluated the disbalance of pro- and anti-oxidants after CA in the plasma during the early phase after resuscitation. This disequilibrium favors the prooxidants and is associated with increased levels of downstream oxidative stress-induced end-products, which the body’s antioxidant capacity is unable to directly mitigate. Here, we suggest that circulating plasma is a major contributor to oxidative stress post-CA and its management requires substantial early intervention for favorable outcomes.

## Introduction

Cardiac arrest (CA) is a global injury that results in impaired circulation of blood throughout the body, depriving all tissues of oxygen as well as other essential metabolites needed for basic cellular respiration (Kim et al. 2016; Kalogeris et al. 2012). The series of downstream effects of CA, collectively termed post-CA syndrome, is expansive, ranging from myocardial stunning to systemic inflammation to cell death contingent upon the patient surviving CA (Neumar et al. 2008; Stub et al. 2011). An important contributor to the varied clinical profile of early post-cardiac arrest is the impaired reintroduction of oxygen to ischemic tissues (Neumar et al. 2008). The impact of cardiac arrest is two-fold: initial injury due to the deprivation of oxygen followed by injury due to the replenishment of oxygen with resuscitation via the implementation of clinical measures such as CPR (Rea et al. 2008). This phenomenon is known as ischemia–reperfusion injury and is experienced by tissues simultaneously (Patil et al. 2015; Verma et al. 2002). In this context, the flooding of oxygen into ischemic tissues with impaired respiratory machinery is harmful (Verma et al. 2002). Given the increasing implication of oxidative species in the pathophysiology of various disease states such as diabetes, hypertension, and hyperlipidemia, it is worth investigating their role in the deleterious sequelae of CA (Volpe et al. 2018; Togliatto et al. 2017; Touyz and Briones 2011; Amiya 2016).

Reactive oxygen species (ROS) are byproducts of cellular respiration as orchestrated by the mitochondria and electron transport chain, among others, and play a significant role in normal physiology (Kalogeris et al. 2012; Touyz and Briones 2011; Onukwufor et al. 2019). ROS is a key player in signaling cascades that result in the regulation of inflammatory pathways or cell migration and proliferation (Weidinger and Kozlov 2015; Schieber and Chandel 2014). However, the physiologic role of ROS must be maintained to balance its beneficial effects with its potential for damage via destruction of membranes and surrounding biomolecules (Rahal 2014; Pizzino et al. 2017). In addition to ROS, reactive nitrogen species (RNS), such as nitric oxide and peroxynitrite, and others, contribute to oxidative stress (Dedon and Tannenbaum 2004). These species can propagate damage via similar mechanisms, such as the oxidation of proteins and DNA mutation (Adams et al. 2015). Outside of the mitochondria, there are other ways of generating oxidants. Xanthine oxidase-mediated reduction of oxygen generates superoxide anion and hydrogen peroxide (Battelli et al. 2016). Plasma levels of xanthine oxidase may be increased after proinflammatory insults that can facilitate damage at distant sites. Neutrophils create oxidative bursts as a mechanism of eliminating bacteria via NADPH oxidase or myeloperoxidase (Dedon and Tannenbaum 2004; Panday et al. 2015).

Altogether, the various compounds that contribute to production of reactive oxygen species fall under the umbrella of prooxidants (Rahal 2014). Under normal physiologic conditions, an equilibrium must be established between prooxidants and antioxidants for metabolic and physiologic activity to continue without causing excessive oxidative damage. To achieve this, tissues are equipped with various players, such as superoxide dismutase, catalase, and the glutathione system, to appropriately manage these reactive species and limit their pathologic roles (Kurutas 2016; Younus 2018; Nandi et al. 2019; Lushchak 2012). Reperfusion injury affects the metabolic environment of major organs such as the brain, heart, kidney, and liver, which is one of the major causes of the high mortality rate associated with CA (Virani et al. 2020; Choi 2019). An important component that must not be ignored in this global picture is the role of plasma. Plasma is a conduit for metabolic, oxidative, and inflammatory interaction among organs via the exchange of metabolites, electrolytes, cytokines, and hormones (Torell 2015). This results in the utilization of the vascular system as a transport medium for any damaging oxidative species to eventually affect distant organs that may themselves be struggling with ROS overload. An analysis of the plasma can reflect the disruptions in oxidant-antioxidant equilibrium occurring in the rest of the body. Therefore, the purpose of this study is to understand and better characterize the interplay between the pro- and anti-oxidants found in plasma during the early phase of resuscitation after cardiac arrest to further elucidate CA pathophysiology.

## Materials and methods

### Animal surgical protocol

**Fig. 1:**
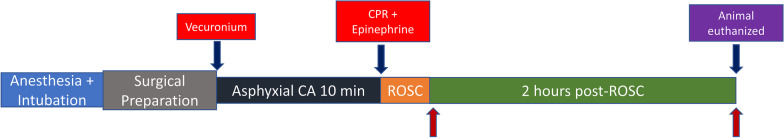
Experimental schematic for asphyxial cardiac arrest (CA) 10 min followed by cardiopulmonary resuscitation (CPR) and successful achievement of return of spontaneous circulation (ROSC). Blood was drawn after ROSC and at 2 h post-ROSC as shown by the red arrows

The experimental protocol was approved by the Institutional Animal Care and Use Committee of the Feinstein Institutes of Medical Research (2017-033). Adult male Sprague–Dawley rats (n = 12; 517.92 ± 18.68 g; Charles River Production, Wilmington, MA) were maintained under a 12-h light/dark cycle with free access to food and water. The animal surgical procedures for the asphyxia-induced CA rat model were conducted using established methods (Kim et al. 2014, 2015, 2016; Han et al. 2010; Kuschner et al. 2018) and are summarized in Fig. 1. Briefly, rats were anesthetized using 4% isoflurane and placed on a ventilator post-intubation. After successful cannulation of the left femoral artery and vein, heparin (300 U; 0.3 mL) was administered via the femoral vein. Physiologic parameters were recorded at baseline for 10 min and 2 mL/Kg of vecuronium was administered via the left femoral venous catheter. Asphyxia was induced by disconnecting the animal from the ventilator. Within 3.52 ± 0.18 min, the mean arterial pressure fell to below 20 mmHg, our predesigned definition of cardiac arrest (Han et al. 2010). Following 10 min of CA, CPR was initiated by chest compressions and ventilation with 100% oxygen was resumed. Epinephrine (0.2 mg/Kg) was administered within 20 s after the initiation of CPR. All rats achieved return of spontaneous circulation (ROSC) in approximately 1.18 ± 0.12 min after the initiation of CPR. Animals were connected to the ventilator and hemodynamic parameters were recorded up to 2 h post-resuscitation. Blood was withdrawn from the left femoral artery catheter immediately after ROSC was achieved and at 2 h post-ROSC. After 20 min post-ROSC, oxygen concentration was decreased from FiO_2_ 1.0 to 0.3. Plasma was isolated from whole blood via centrifugation for 10 min at 1000×*g* and immediately frozen and stored at −80 °C to minimize freeze–thaw cycles. Rats were then euthanized. Blood was drawn from rats who did not undergo CA to serve as control blood; animals were deeply anesthetized, and blood was withdrawn before being euthanized (Choi 2019).

### Prooxidant determination

The prooxidant detection was accomplished using colorimetric assays conducted according to manufacturer’s instructions with minor modifications: Amplex Red (Invitrogen, Carlsbad, CA), Dihydroethidium (DHE; Cayman Chemical, Ann Arbor, MI), Nitrate/Nitrite Concentration (Cayman Chemical, Ann Arbor, MI), and Xanthine Oxidase Activity (BioVision Inc., Milpitas, CA). The Amplex Red assay is used to measure a variety of oxidation species comprising of, but not limited to, hydrogen peroxide, peroxynitrite, lipid peroxides, and others (Debski et al. 2016; Miwa et al. 2016). Briefly, for Amplex Red oxidation assay, hydrogen peroxide standard curve with final concentrations ranging from 0 μM to 100 μM was generated. Undiluted plasma samples were used. Absorbance readings were then taken every 30 min for 3 h at a wavelength of 560 nm with a Modulus Microplate Reader Model 9300–062 (Turner Biosystems, Sunnyvale, CA) and the rate of Amplex Red oxidation was determined. All other assays were read using the Spark microplate reader (Tecan, Männedorf, Switzerland).

Dihydroethidium oxidation was performed using a modification of an established method (Nazarewicz et al. 2013). Briefly, DHE was diluted in dimethyl sulfoxide (DMSO; Sigma Aldrich, St. Louis, MO) to make a stock of 32 mM, which was further diluted to 100 μM in 50 mM sodium phosphate buffer (pH 7.4) and combined with plasma 1:1 by volume and read in kinetic mode at 5 min intervals for 60 min with excitation at 480 nm/emission at 580 nm. Inhibition of xanthine oxidase was performed with a final concentration of 50 nM of febuxostat in DMSO, which is well above the half-maximal inhibitory concentration (IC_50_) of 2.2 nM (Osada et al. 1993). The Nitrate/Nitrite Concentration assay was performed using plasma samples concentrated with a 10 kDa Amicon concentrator (MilliporeSigma, Burlington, MA) according to manufacturer’s protocol. The filtrate was then used for the assay, which was read at 540 nm. The Xanthine Oxidase activity assay was performed using undiluted plasma and read at 570 nm at 2 min intervals for 30 min. The iron concentration assay (Bioassay Systems, Hayward, CA) was run according to manufacturer’s protocol and read at 590 nm.

### Antioxidant determination

The antioxidant detection was accomplished using four major antioxidant mechanisms that include enzymatic and nonenzymatic reduction of reactive oxidants. The two enzymatic antioxidants measured were Catalase activity and Superoxide Dismutase activity (Cayman Chemical, Ann Arbor, MI). The two nonenzymatic antioxidant assays performed were Total Antioxidant Capacity (TAC; Cayman Chemical, Ann Arbor, MI), and Ferric Reduction Antioxidant Potential (FRAP; Bioassay Systems, Hayward, CA). The assays were conducted according to manufacturers’ instructions with minor modifications. Superoxide Dismutase Activity and Total Antioxidant Capacity were measured with 1:5 plasma dilution and read at 440 and 750 nm, respectively. FRAP was measured with 1:2 plasma dilution and read at 590 nm. Catalase activity was measured without plasma dilution and read at 540 nm. All dilutions were performed in accordance with respective manufacturers’ protocols.

### Oxidation products determination

Oxidants can react with endogenous proteins, lipids, and other materials to form oxidation products. Protein carbonylation was detected using a protein carbonyl colorimetric assay (Cayman Chemical, Ann Arbor, MI) and measured at 370 nm. The detection of lipid peroxides in the form of malondialdehyde (MDA; Cayman Chemical, Ann Arbor, MI) measured at 535 nm, and 4-Hydroxyl-2-noneal (4-HNE) and neuron specific enolase (NSE) (MyBioSource, Inc., San Diego, CA) measured at 540 nm and 450 nm, respectively. Protein nitrotyrosine concentration (StressMarq Biosciences Inc., Victoria, BC, Canada) was measured with 1:2 plasma dilution and read at 450 nm. The assays were conducted according to manufacturer’s instructions without other modifications not mentioned.

### Statistical analyses

All data are presented as mean ± standard error of the mean (SEM) with statistical significance determined using (1) one-way analysis of variance (ANOVA) followed by Tukey's multiple comparisons test, or (2) paired/unpaired two-tailed Student’s t-test or Mann–Whitney U test, as appropriate after determination of parametric distribution. All statistical analyses were conducted using GraphPad Prism 9.0 (GraphPad Software Inc., La Jolla, CA). P values of < 0.05 were considered statistically significant.

## Results

### Physiologic outcomes after cardiac arrest

**Fig. 2:**
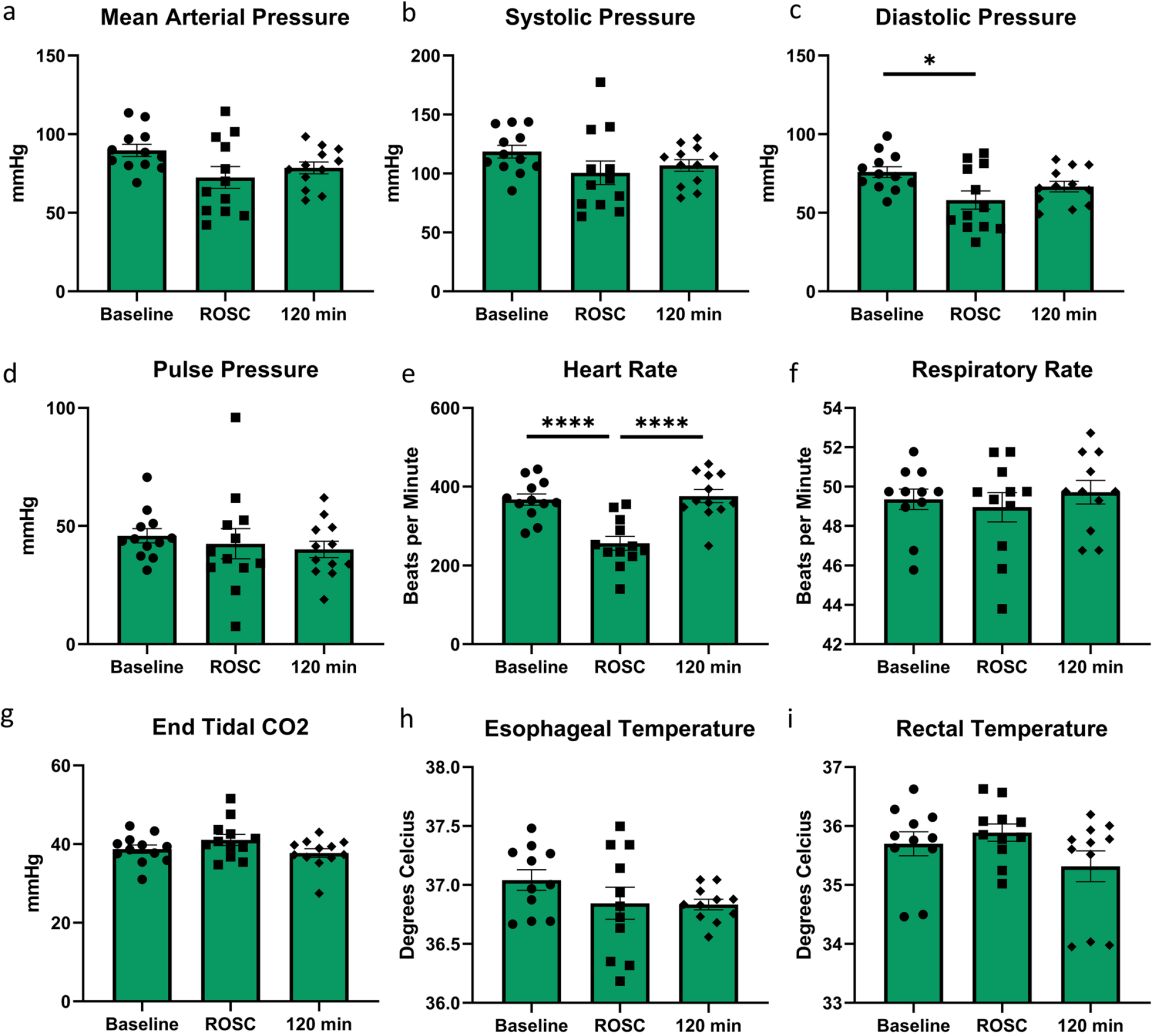
Physiologic parameter comparison. No major changes in the physiologic parameters (esophageal and rectal temperature, respiratory rate, end-tidal CO_2_, mean arterial pressure, systolic pressure, and pulse pressure) were observed except for the diastolic pressure (**c**) between Baseline and ROSC (P < 0.05) and the heart rate (**e**) between Baseline to ROSC and ROSC to 2 h post-ROSC (P < 0.0001, respectively). Data represented as Mean ± SEM.*p < 0.05 and ****p < 0.0001; ROSC, return of spontaneous circulation

Physiologic data was collected to ensure consistency under the experimental conditions. No significant differences were found among baseline, ROSC, and 2 h post-ROSC for the following physiologic data parameters collected: esophageal and rectal temperature, respiratory rate, end-tidal CO_2_, mean arterial pressure, systolic pressure, heart rate, and pulse pressure (Fig. 2). As predicted, given the nature of cardiac arrest, a significant decrease in heart rate is seen between baseline and ROSC group (P < 0.0001), while a significant increase in heart rate is seen between ROSC and 2 h post-ROSC groups (P < 0.0001). A significant decrease in diastolic pressure is seen between baseline and ROSC groups (P < 0.05). These changes signify the toll of CA on the cardiovascular physiology that are expected in a 10 min CA rat model.

### Prooxidant capacity increased during the early phase after resuscitation

**Fig. 3:**
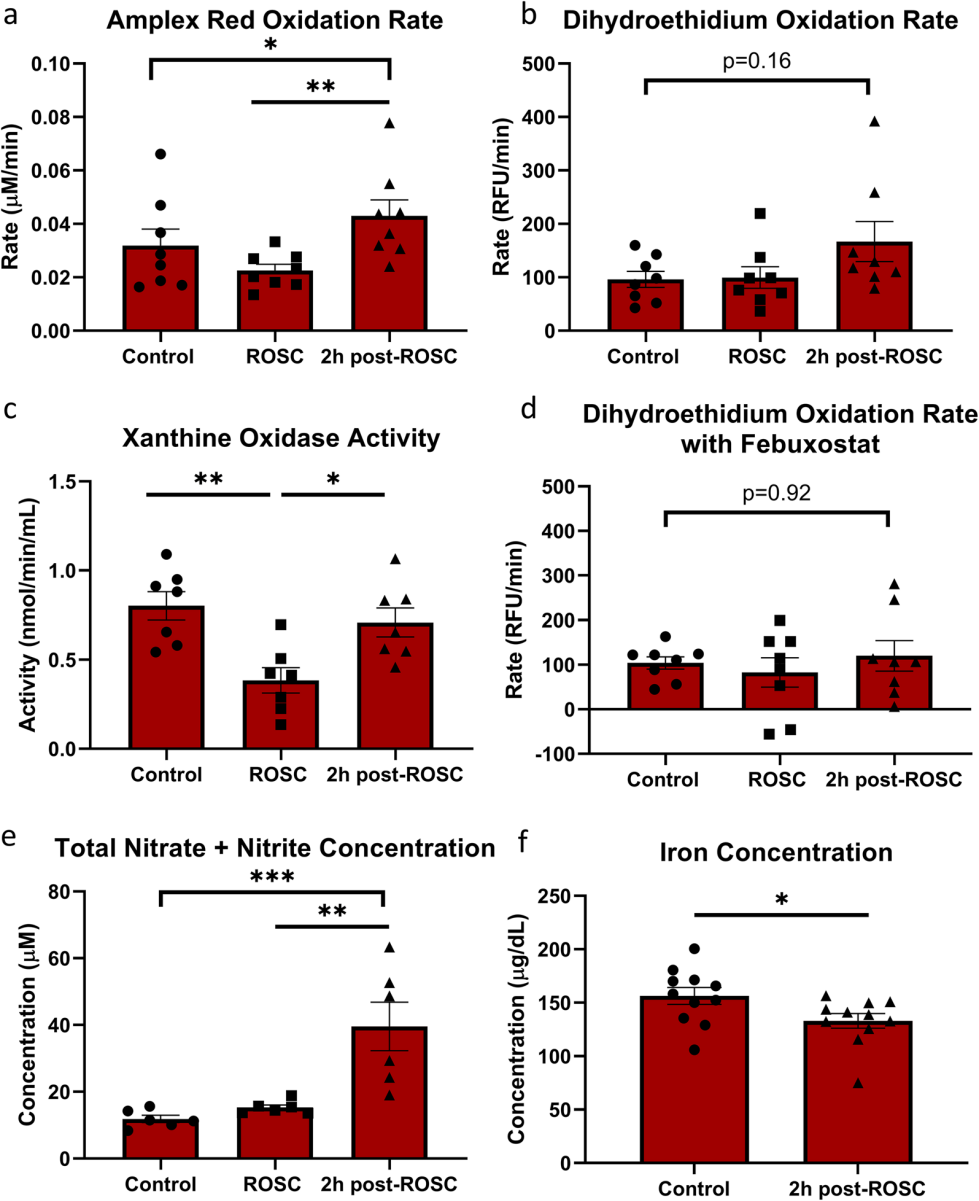
**Increased prooxidant levels after CA**. **a** Rate of Amplex Red oxidation in rat plasma is decreased at ROSC and substantially increased 2 h post-ROSC. **b** Rate of dihydroethidium oxidation shows an increasing trend at 2 h post-ROSC. **c** Xanthine oxidase activity is significantly decreased at ROSC but increases 2 h post-ROSC. **d** Rate of dihydroethidium oxidation is unchanged at 2 h post-ROSC as compared with control with the addition of the xanthine oxidase inhibitor febuxostat. **e** Total nitrate and nitrite concentration is significantly increased at 2 h post-ROSC. **f** Plasma iron concentration significantly decreased at 2 h post-ROSC. *p < 0.05, **p < 0.01, and ***p < 0.001; ROSC, return of spontaneous circulation

Prooxidants can be pathological as observed in tissue injury after CA, but also have significant physiological roles in the body (Kalogeris et al. 2012; Touyz and Briones 2011; Onukwufor et al. 2019). A commonly used assay to detect hydrogen peroxide is Amplex Red. However, under the influence of a variety of other oxidants, such as radicals, nitrites, and even enzymes, the compound Amplex Red is oxidized to its fluorescent counterpart, resorufin. Due to the lack of specificity for any one oxidant, this assay serves as a surrogate measurement of overall oxidative stress that includes ROS, their derivatives, and oxidative enzymes. Although statistically nonsignificant, the rate of Amplex Red oxidation was almost cut by approximately 25% at ROSC (Fig. 3a). A significant increase was observed in the oxidation rate following ROSC (P < 0.01), which was greater than the rate observed in control plasma (P < 0.05). DHE oxidation was also evaluated in plasma because it is oxidized by superoxide and has more specificity as compared to Amplex Red (Nazarewicz et al. 2013). Plasma obtained 2 h post-ROSC, demonstrated an increased rate in its DHE oxidation as compared to control suggesting increased superoxide generation post-ROSC (P = 0.16; Fig. 3b). As a major contributor to superoxide generation, we measured xanthine oxidase (XO) activity. XO activity was significantly decreased between control and ROSC (P < 0.01) and then significantly increased at 2 h post-ROSC (P < 0.05; Fig. 3c). Next, we decided to evaluate the oxidation rate of DHE with the addition of febuxostat, a potent XO inhibitor. The oxidation rate of DHE was largely unchanged with the addition of febuxostat in plasma at control or at ROSC. However, the initial increased trend observed in DHE oxidation rate at 2 h post-ROSC was substantially reduced to the levels of control plasma after the addition of febuxostat, highlighting the role of XO in plasma oxidative stress as a function of CA (Fig. 3d).

A contributor to the increased oxidation rate of Amplex Red is peroxynitrite, which is a subsidiary in the nitrate/nitrite/nitric oxide pathway. Our analysis of total plasma nitrate and nitrite concentration demonstrated no major change in the total concentration of nitrates and nitrites during the ischemic phase of CA. However, following 2 h post-ROSC there is a substantial increase in the total concentration of nitrates and nitrites as compared to ROSC and control levels (P < 0.01 and P < 0.001, respectively; Fig. 3e). Increased nitrate/nitrite concentration suggests an increase in reactive nitrogen species (RNS) 2 h after CA and resuscitation, which is a subset of oxidative stress mediators that work in a similar fashion as ROS to cause damage (Dedon and Tannenbaum 2004; Weitzberg et al. 2010). As a major catalyst of oxidative stress, we measured the plasma concentrations of free iron that would participate in the Fenton reaction to produce damaging hydroxyl species. We observed a decrease in iron concentration at 2 h post-ROSC compared with control levels (P < 0.05; Fig. 3f). It may seem that lower iron content may help to limit the amount conversion of H_2_O_2_ to the more damaging hydroxyls (^·^OH). However, recent evidence has suggested that mice post-CA displayed a flux of iron from the plasma into tissues post-CA that results in downstream organ injury through ferroptosis pathways (Miyazaki et al. 2020).

### Antioxidant capacity decreased during the early phase after resuscitation

**Fig. 4:**
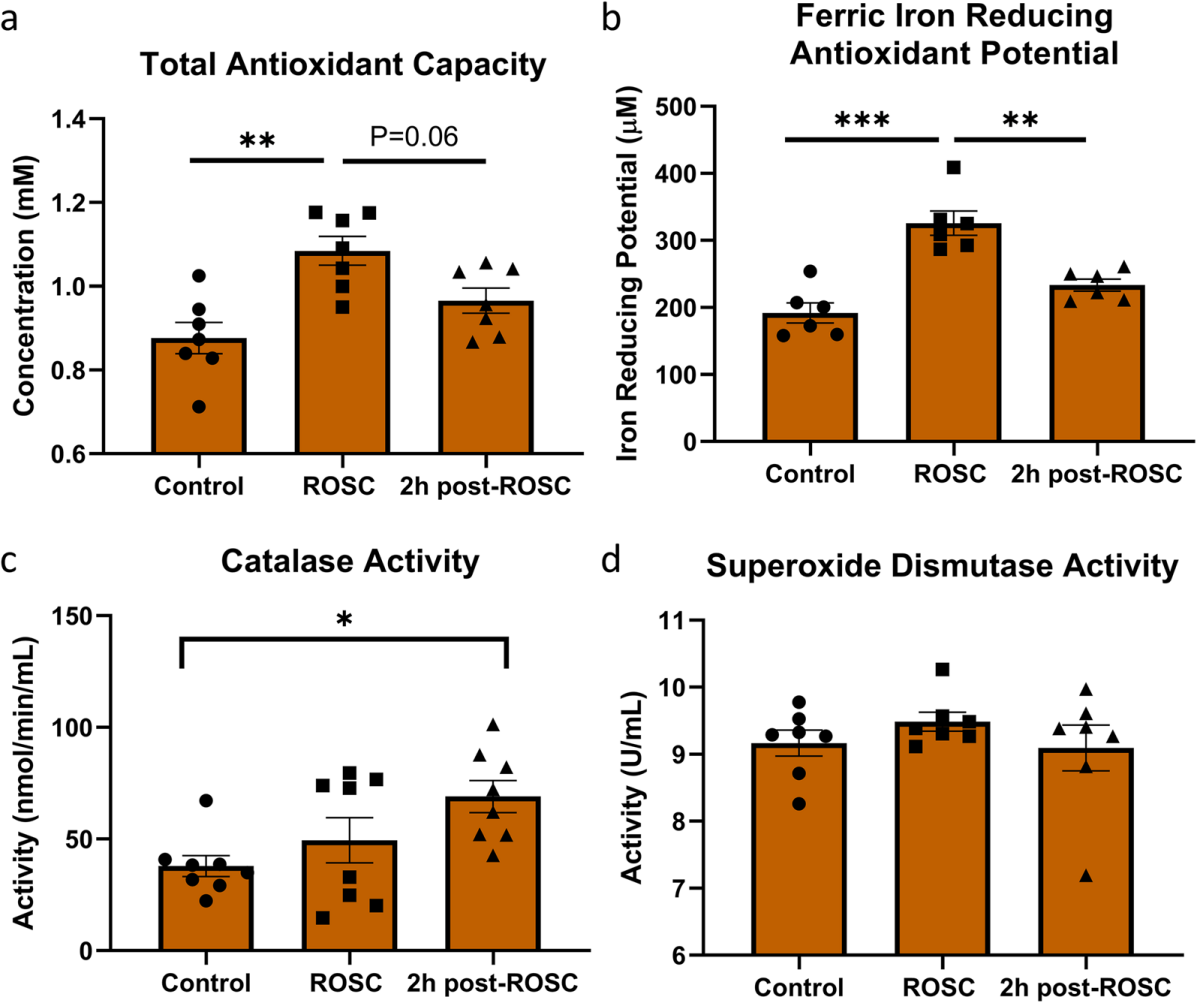
Increased antioxidant levels during ischemia. **a** Total Antioxidant Capacity is increased at ROSC in rats. **b** Ferric Iron Reducing Antioxidant Potential is increased at ROSC in rats. **c** Significantly increased catalase activity is seen only when compared between 2 h post-ROSC and control in rats. **d** No changes are observed in superoxide dismutase activity at ROSC and 2 h post-ROSC in rats. *p < 0.05, **p < 0.01, and ***p < 0.001; ROSC, return of spontaneous circulation

Total antioxidant capacity (TAC) is used as an estimation of the multitude of antioxidants available in a given system. There was a substantial increase in TAC after ROSC (P < 0.01), but a dramatic decrease from ROSC to 2 h post-ROSC (P = 0.06; Fig. 4a). There was no significant difference in TAC at 2 h post-ROSC as compared with control. Ferric iron reduction antioxidant potential (FRAP) is another common assay utilized to measure antioxidants in a sample by the ability to reduce exogenously added ferric iron to ferrous iron. Similar to TAC, FRAP is significantly increased at ROSC followed by a substantial decrease between ROSC and 2 h post-ROSC (P < 0.001 and P < 0.01, respectively; Fig. 4b). There was no difference observed in FRAP at 2 h post-ROSC compared with control. Analysis of a major antioxidant enzyme, catalase, that neutralizes H_2_O_2_, shows increased catalase activity at 2 h post-ROSC when compared with control, albeit the rate of increase not being substantially large (P < 0.05; Fig. 4c). Interestingly, no significant changes are observed in superoxide dismutase activity among the three time points measured (Fig. 4d).

### Oxidation products increased during the early phase after resuscitation

**Fig. 5:**
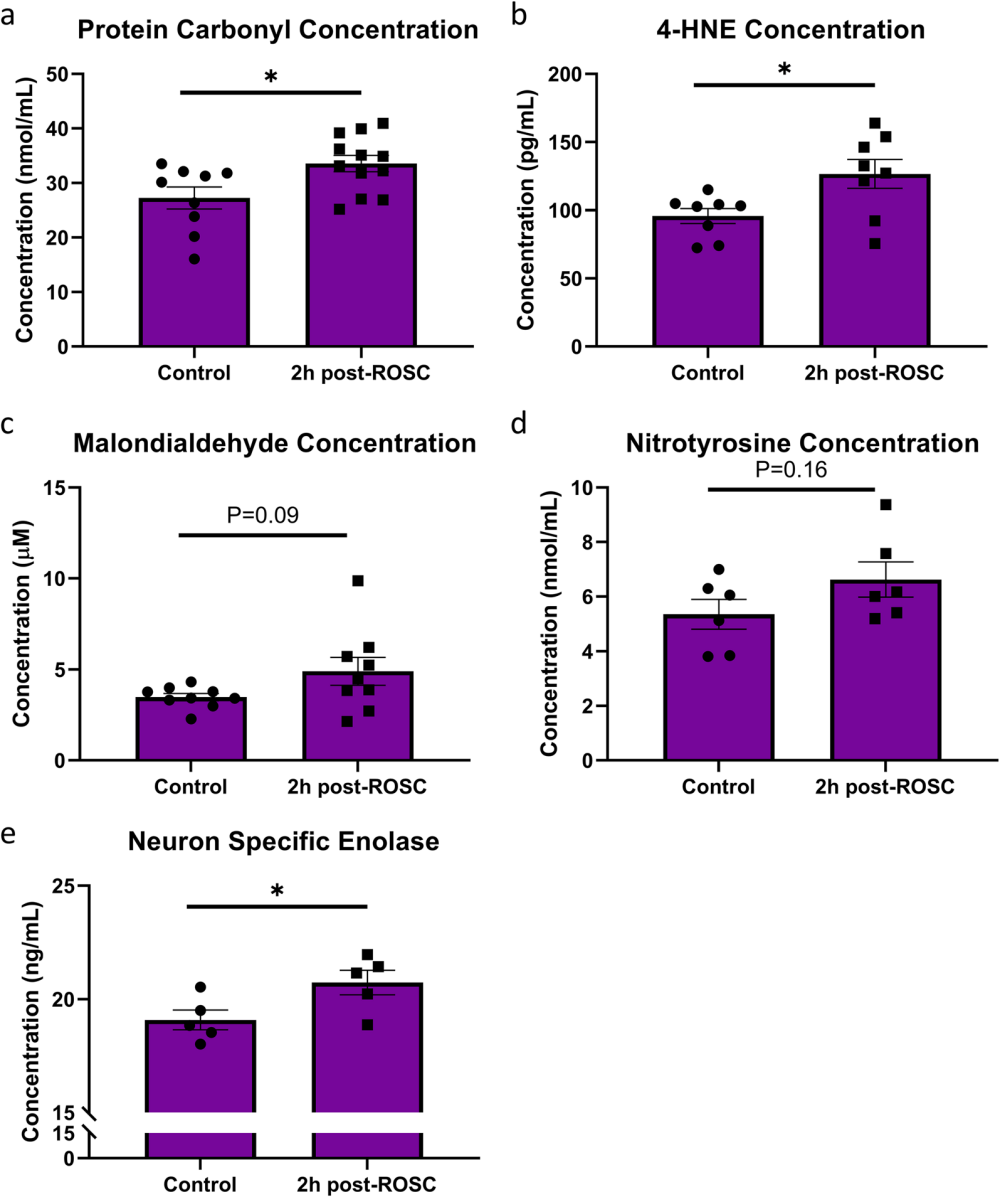
Increased levels of oxidative damage products. **a** A significant increase in protein carbonylation is observed 2 h post-ROSC in rats. **b** 4-HNE concentration significantly increases 2 h post-ROSC in rats. Malondialdehyde (**c**) and nitrotyrosine (**d**) concentrations trend upward 2 h post-ROSC, while neuron specific enolase (**e**) is significantly increased post-ROSC. *p < 0.05, **p < 0.01, and ***p < 0.001; ROSC, return of spontaneous circulation; 4-HNE, 4-Hydroxyl-2-noneal

Oxidation products are produced by various reactive oxygen and nitrogen species that damage surrounding cellular and extracellular components, such as lipids and proteins. A significant increase was observed in protein carbonyl concentration after 2 h post-ROSC (P < 0.05; Fig. 5a). A similar significant increase was also observed in 4-Hydroxyl-2-noneal (4-HNE) concentration 2 h post-ROSC (P < 0.05; Fig. 5b). A non-statistically significant increasing trend after 2 h post-ROSC was observed for malondialdehyde (MDA) (p = 0.09; Fig. 5c) and nitrotyrosine concentrations (P = 0.16; Fig. 5d). An increase in NSE, which is not a direct oxidation product, but signifies end-organ damage in the brain, was observed 2 h post-CA in the plasma (Fig. 5e). Overall, plasma measurement of various oxidation end-products shows an increasing trend post-CA.

## Discussion

Cardiac arrest results in ineffective perfusion of oxygen and other metabolites to tissues during ischemia; resuscitation produces a dramatic rise in oxygen content in various tissues that were attempting to adapt unsuccessfully to suboptimal levels of oxygen utilization (Shoaib and Becker 2020). Given the crucial role of oxygen in cellular respiration and the variety of measures needed to keep harmful radicals in check, this study investigates how lack of oxygen during ischemia followed by the replenishment of oxygen after resuscitation affects the delicate balance between prooxidants and antioxidants in plasma. Overall, our results demonstrate that this homeostatic balance is substantially affected post-CA and significantly favors prooxidant production following resuscitation. This finding may be one of the physiological explanations behind organ injury and poor outcomes during the early phase of resuscitation that affects the entire body as the plasma is circulating around. The plasma is not inert and it actively participates in global oxidative stress.

Two general trends are observed throughout the course of cardiac arrest from ROSC to 2 h post-ROSC. Firstly, prooxidants in the plasma that include enzymes, such as XO or NADPH oxidases, as well as molecular oxidants such as hydrogen peroxide, significantly increase after ROSC up to the measured 2 h post-ROSC (Fig. 3). During the ischemic phase of CA, the lack of a constant influx of oxygen produces an environment that is rapidly desaturated of oxidants as we observed through various surrogate markers. However, after resuscitation, the flood of oxygen into the system results in substantially increased prooxidants, with the potential to continue increasing beyond our endpoint of measurement. Xanthine oxidase has the capacity to generate superoxide radicals, which in turn can produce hydrogen peroxide that can participate in ischemia–reperfusion injury and contribute to the oxidation of Amplex Red and DHE (Battelli et al. 2016; Pacher et al. 2006). In fact, the decrease of DHE rate post-CA using febuxostat highly supports the role of XO in generation of a prooxidant environment in the plasma. Although previously considered as simple, less active end products of nitric oxide generation, nitrates and nitrites have been implicated in more complex physiology that can result in the protective modulation of cellular metabolism, vascular regulation, and cell signaling; however, in the setting of ischemia–reperfusion injury, the nitrate/nitrite increase can facilitate the conversion to nitric oxide and peroxynitrite and begin an oxidative cycle contributing to tissue damage (Dedon and Tannenbaum 2004; Weitzberg et al. 2010; Warner et al. 2004). The substantial increase observed 2 h post-ROSC suggests that the deleterious effects of nitrate/nitrite may outweigh any beneficial vasodilatory effects during the initial ischemic phase. This results in increased RNS-mediated damage. Furthermore, the substantial decrease in plasma free iron may also indicate that post-CA rats have a net iron efflux distally from the vascular system that can potentiate oxidative damage in individual tissues.

Our second general trend is that the plasma antioxidant capacity that includes a variety of antioxidants, both enzymatic and molecular, increases during the ischemic phase of cardiac arrest as measured immediately post-ROSC (Fig. 4). The post-ROSC measurements are the closest approximations to the pathophysiology of the ischemic phase of CA as it is technically challenging to withdraw blood when there is no heart function. Our results do not directly differentiate the source of increased antioxidant capacity. It could be a function of increased activity of antioxidant enzymes or generation of antioxidant molecules, but it could also be the result of ischemia that lowered oxygen concentration and consequentially decreased oxidants. It seems more likely the latter may be the primary factor as the body’s synthesis mechanisms may not actually be working optimally to produce a net generation after CA. Regardless, after the initial increase, the antioxidant capacity trends downward with time following resuscitation.

Our data suggests that oxidative damage brought by resuscitation is enough to match the antioxidant defenses that may have accumulated and/or not consumed during ischemia and is also enough to exceed and overwhelm it. In accordance with recent proposal of decreasing hyperoxic damage post-CA (McKenzie and Dobb 2018), we experimentally designed to mitigate damage by hyperoxia post-ROSC by decreasing FiO_2_ to 0.3. However, prooxidants were substantially increased after resuscitation. Together, this culminates into the oxidative end-products observed in the plasma. Lipids of surrounding cellular or organelle membranes along with those found freely in plasma can be attacked by free radicals and oxidative enzymes, resulting in degradation products such as lipid peroxides and aldehydes (Ayala et al. 2014; Pizzimenti et al. 2013). Polyunsaturated fatty acids (PUFAs) in particular are vulnerable as a result of inherently increased electron instability from carbon–carbon double bounds as opposed to their saturated counterparts (Varela-Lopez et al. 2015). Given the integral part of PUFAs in the fluidity and proper functionality of cellular and organelle membranes (Harayama and Shimizu 2020), the effect of reactive oxygen species can be detrimental. However, lipid peroxidation is particularly important as the products of these reactions, such as MDA and 4-HNE, are toxic (Ayala et al. 2014; Dalleau et al. 2013). Not only can these products be directly harmful, but they can also participate in and further propagate the oxidant generation system. For example, 4-HNE has the ability to uncouple mitochondria, participate in protein carbonylation, and serve as a secondary messenger in apoptosis and inflammatory pathways (Breitzig et al. 2016). MDA can similarly damage the cellular environment resulting in the production of protein and DNA adducts (Ayala et al. 2014). Increased low molecular weight chelated iron accumulation in the brain was directly associated with increased MDA formation following resuscitation in dogs (Nayini et al. 1985), validating our findings of increased lipid peroxides and decreased iron in the plasma. In this study, the significant increase in 4-HNE and upward trend in MDA 2 h post-ROSC strongly suggests that the oxidative stress inflicted on major organs by cardiac arrest is indeed reflected in the plasma. Similarly, both free radicals and lipid peroxides can damage proteins. Proteins carbonylation via ROS and nitrosylation via RNS correlate with higher oxidative stress (Wong et al. 2010; Suzuki et al. 2010; Shahani and Sawa 2012). We see an increase in protein carbonylation nitrotyroslation post-ROSC, establishing a consistent theme in the plasma of ischemia–reperfusion injury.

Given the global impact of cardiac arrest, it is necessary to evaluate the utility of plasma in representing end-organ damage, particularly in the brain. NSE is expressed on neurons, neuroendocrine cells, and glial cells. NSE levels are major influencers in the cellular decision to degenerate or proliferate and are involved in propagating neuroinflammation when indicated (Haque et al. 2018). It has been used to correlate oxidative damage to the prediction of neurological outcomes of unconscious CA patients and in perinatal hypoxic-ischemic brain injury (Wiberg 2017; Vasiljevic et al. 2012). Increased plasma NSE levels at 2 h post-ROSC were observed, suggesting that the brain is indeed suffering oxidative damage. The ability of tissues to maintain oxidative damage within homeostatic limits is compromised by the reperfusion phase of cardiac arrest. Given the role of plasma as a conduit throughout the body for any and all molecules, the substantial increase in oxidation products in the plasma is highly reflective of parallel oxidative stress in the surrounding tissues.

**Fig. 6:**
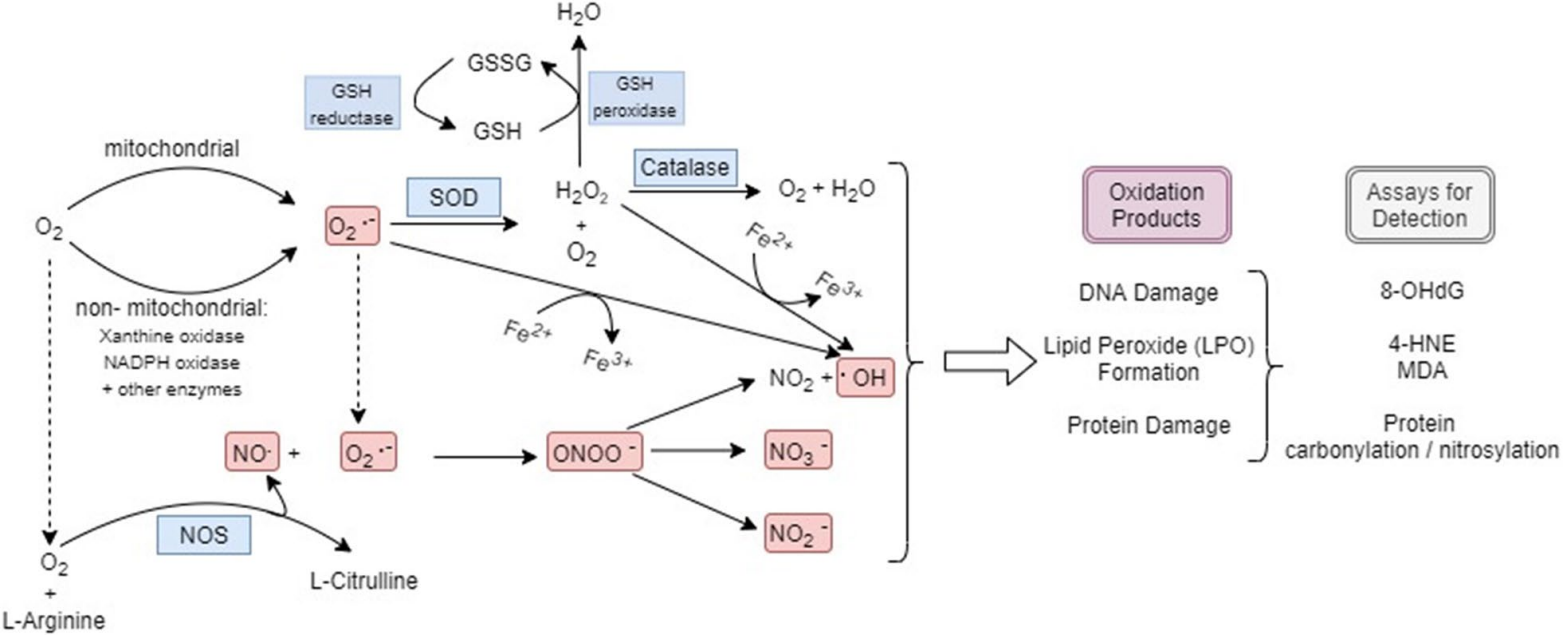
Diagrammatic representation of major Prooxidant and Antioxidant Pathways as well as the end-products after ischemia–reperfusion injury post-CA. Red represents prooxidants; blue represents antioxidants. 4-HNE, 4-Hydroxyl-2-noneal; 8-OHdG, 8-hydroxy-2’-deoxyguanosine; GSH, glutathione; GSSG, glutathione disulfide; MDA, malondialdehyde; NOS, nitric oxide synthase; SOD, superoxide dismutase

The described results represent an interpretation of the complex physiology that happens under normal circumstances as it focuses on a limited selection of key players in the prooxidant and antioxidant systems. Figure 6 depicts the interplay of major prooxidant and antioxidant pathways in the setting of ischemia–reperfusion following CA. When viewed in the larger context of post-CA syndrome, it is understood that oxidative damage is one of the many factors leading to increased morbidity (Neumar et al. 2008). This is a possible explanation for why antioxidant vitamin supplementation has not been the panacea of CA treatment as this only targets a small part of the entire oxidant-antioxidant system (Ye et al. 2013; Spoelstra-de Man et al. 2018; Gardner et al. 2020). In contrast to these antioxidant targeted trials is the use of hypothermia, which has been shown to reduce oxidative damage in the post-CA state (Hackenhaar et al. 2017; Dohi 2013). It is hypothesized that hypothermia globally decreases metabolism and thereby reduces the formation of radicals. However, hypothermia is not without its own deleterious effects (Soleimanpour et al. 2014). Furthermore, recent studies have highlighted a potential increase in oxygen consumption without a proportional increase in carbon dioxide generation producing a lowered respiratory quotient, signifying reduced mitochondrial efficiency (Shinozaki et al. 2018). It is highly likely that post-CA, the mitochondria are not primed to fully utilize the incoming oxygen (Shoaib and Becker 2020) and the proportion of non-mitochondrial oxygen consumption and/or the proportion of electron leak from the mitochondria is enhanced after injury contributing to increased imbalance between pro- and anti-oxidants, as we observed. This lack of primed oxygen usage can be reflected in the plasma, which is then forced to carry a higher oxidative burden leading to the plasma itself being a carrier of injury-causing substrates.

Similar to all experimental studies, a limitation of this study is the controlled environment of CA and resuscitation in our rat model that does not directly reflect the variability of human CA. Few patients are healthy prior to CA as most patients have comorbidities that complicate the issue of disequilibrium post-CA. The widely accepted models of rodent CA utilize healthy rats prior to inducing CA. Despite the lack of comorbidities and difference in mode of resuscitation in humans vs. our rat model, we have previously shown substantial similarities in the metabolic change in both human and rat plasma (Shoaib et al. 2020), providing support that our findings are still translatable to study humans who have a plethora of medical conditions. Measuring individual oxidative species is a challenging task because experimentally measuring fleeting species is not facile, many species are easily interconvertible, and there is contribution from both mitochondrial and non-mitochondrial sources. As such, we employed the use of direct and indirect surrogates. In particular, glutathione is an important part this system, as it is able to directly react with reactive oxygen and nitrogen species and eliminate them (Lushchak 2012). However, despite numerous attempts, we were unable to quantify the activity of the glutathione system. Furthermore, we were unable to detail the oxidant-antioxidant imbalance past 2 h post-ROSC. It is highly likely that homeostasis may be established, but it is still unknown at what time after ROSC that would occur, and the effects of the damage incurred this early after resuscitation. Despite these limitations, our data provides more details regarding the prooxidant and antioxidant disbalance post-CA. A single administration of one antioxidant to curtail this plethora of dysfunction, which has been the current trend in therapeutic approaches for CA, is insufficient to prevent the significant injury that leads to poor outcomes in CA patients. This necessitates a multidimensional use of antioxidant therapies that take into account the plasma compartment for ROS along with the tissue compartments and that are sustained during resuscitation and beyond to appropriately combat the increasing trend of prooxidant production seen after ROSC. Taken together, this analysis of the plasma after rodent cardiac arrest and resuscitation clearly demonstrates the major disruptions in homeostatic mechanisms normally used to balance physiologic levels of oxidative species.

## Conclusion

The increase in rates and activities of various mediators observed in our ex-vivo measurements support the plasma as an entity of oxidative stress regardless of its direct interaction with the other tissues. The disequilibrium between plasma oxidative and antioxidative capacity observed after resuscitation means that the increased oxidation mediators overwhelm the defensive molecular and enzymatic capacity of the plasma that then circulates to individual tissues and not only contributes to, but also further extracts from the tissues prooxidants that contribute to global injury in a non-linear mechanism.

## Data Availability

The data that support the findings of this study are available from the corresponding author upon reasonable request.

## Abbreviations

4-HNE: 4-Hydroxyl-2-noneal
CA: Cardiac arrest
CPR: Cardiopulmonary resuscitation
DHE: Dihydroethidium
DMSO: Dimethyl sulfoxide
FRAP: And Ferric Reduction Antioxidant Potential
IC_50_: Half-maximal inhibitory concentration
MDA: Malondialdehyde
NADPH: Nicotinamide adenine dinucleotide phosphate
NSE: Neuron specific enolase
PUFA: Polyunsaturated fatty acids
RNS: Reactive nitrogen species
ROS: Reactive oxygen species
ROSC: Return of spontaneous circulation
XO: Xanthine oxidase
TAC: Total Antioxidant Capacity
